# Efficacy of management of associated dysfunctions on rotator cuff and long head of the biceps: systematic review

**DOI:** 10.1186/s13018-021-02621-0

**Published:** 2021-08-16

**Authors:** Rocio Aldon-Villegas, Veronica Perez-Cabezas, Gema Chamorro-Moriana

**Affiliations:** 1 Department of Physiotherapy, Research Group “Area of Physiotherapy” CTS-305, University of Seville, 41009 Seville, Spain; 2 Department of Nursing and Physiotherapy, Research Group “Empowering Health by Physical Activity, Exercise and Nutrition” CTS-1038, University of Cadiz, 11009 Cadiz, Spain

**Keywords:** Rotator cuff, Shoulder, Biceps, Surgery, Rehabilitation

## Abstract

**Background:**

The important functional role the rotator cuff (RC) and biceps play in the shoulder, the close anatomical relationship between them and the high incidence of injuries require an appropriate multidisciplinary therapeutic approach after a rigorous assessment. The objective is to identify and analyze surgical interventions, whether or not followed by a postsurgical one, of associated dysfunctions on the RC and long head of the biceps (LHB) and their effectiveness in improving shoulder functionality.

**Methods:**

A systematic review based on PRISMA protocol was conducted using PubMed, Web of Science, PEDro, Scopus, CINAHL, and Dialnet until 22 April 2021. The main inclusion criteria were as follows: randomized clinical trials including subjects diagnosed with RC and LHB lesions who had surgical and/not post-surgical treatments. The methodological quality of trials was evaluated by the PEDro scale. Data were shown in 3 pre-established tables: (1)sample data, diagnostic methods, dysfunctions and injury frequency, interventions, outcome measures and results; (2)significance and effectiveness of interventions; and (3)comparison of the effectiveness of interventions.

**Results:**

Eleven studies were selected. The methodological quality of ten of them was assessed as *good* and one *excellent* (PEDro scale). All articles had surgical treatments and ten had postoperative management. All trials used arthroscopy and two open surgery too. Single-row, double-row and transosseous repair were used for RC lesions, while SLAP repair, tenotomy, and tenodesis were applied to LHB injuries. Measured parameters were functionality, pain, Popeye’s sign, strength, range of motion, satisfaction degree, biceps cramping, and quality of life. All approaches in general, surgical plus postsurgical, were always effective to the parameters measured in each study. Seven trials compared tenotomy and tenodesis: four of them obtained statistically significant differences in favor of tenodesis in Popeye’s sign, cramping, satisfaction degree, and/or forearm supination strength; and one, in favor of tenotomy in cramping. All studies measured functionality using functional assessment scales. The most widely used was the Constant Score.

**Conclusions:**

Surgical plus post-surgical interventions in associated dysfunctions on RC and LHB were effective. Tenodesis obtained better results than tenotomy in Popeye’s sign, satisfaction, and forearm supination strength. However, there was no difference regarding biceps cramping.

**Supplementary Information:**

The online version contains supplementary material available at 10.1186/s13018-021-02621-0.

## Introduction

The rotator cuff (RC), which plays a fundamental role in the stability of the shoulder joint complex, allows its optimal functionality by also orienting the upper limb in the three axes of space [1]. It consists of supraspinatus, infraspinatus, teres minor and subscapularis muscles [2], although some authors [3] include the long head of the biceps (LHB) due to its important stabilizing role in the glenohumeral joint [3].

Currently, there is a high index of omalgias, some of them due to both the general involvement of RC and, in particular, the long head of the biceps tendon (LHBT). In relation to the RC, the dysfunction affects 5.7 million Americans of over 60 years of age, accounting for 10% of the population [4]. In fact, said dysfunction has even been increasing in recent decades [5], which also implies an increase in treatments, as it is almost the most prevalent condition treated using orthopedic surgery [6]. On the other hand, LHBT pathologies (tenosynovitis, partial or total tendon tears, subluxations, or dislocations [7]) rarely appear in isolation and are commonly associated with RC [8] due to the close anatomical relationship between the two structures [9].

The frequent involvement of shoulders are the third cause of consultation in primary care and causes pain, stiffness, weakness, or joint instability that results in a detriment in the patient’s quality of life [10]. So much so, that approximately 1% of adult populations in developed countries have shoulder disorders [4]. In half of these, the symptoms persist over a long period of time [11], which leads to a significant consumption of care and socio-economic resources, as well as production losses due to absenteeism. Thus, due to the important functional role the RC and biceps play in the shoulder [1], the close anatomical relationship between them [9], the high incidence of injuries [11], and the high prevalence of orthopedic surgeries that treat such dysfunctions [6]; these structures need to be addressed to obtain appropriate multidisciplinary diagnoses and interventions, i.e., surgical and conservative treatment, the latter including physiotherapy.

Consequently, the recovery rate of the patient will increase, the need for health care will decrease, and labor productivity will improve, which translates into a reduction in health costs.

Therefore, the aim of this systematic review was to identify and analyze surgical interventions, whether or not followed by a postsurgical one, of associated dysfunctions on the rotator cuff and long head of the biceps and their effectiveness in improving shoulder functionality.

## Methods

The method used in this systematic review is based on the PRISMA statement [12].

### Data sources and search strategy

An electronic search of PubMed, Web of Science, PEDro, Scopus, CINAHL, and Dialnet was carried out from inception through April 22, 2021. Mesh terms (Medical Subject Headings) for English language and other terms of interest for frequency of use, or Decs Terms (Descriptores en Ciencias de la Salud) for Spanish database and search strategies are shown in Table 1.

**Table 1:** MeSH and DeCS terms put into groups by mean and search strategy

Terms		Identifier
*“Rotator cuff”*		1
*Biceps* or “long head of the biceps tendon” or “long head of the biceps brachii tendon”		2
Injury* or torn* or wound* or dislocation* or tear*		3
*Shoulder*		4
*“Treatment outcome”*		5
*“Clinical trial”*		6
“Manguito de los rotadores”		7
Bíceps or “porción larga del bíceps”		8
Lesión* or desgarro* or luxación*		9
**Database**	**Search strategy**	**Simplified strategy**
Pubmed	(“*rotator cuff*” or *shoulder*) and (*biceps* or “long head of the biceps tendon” or “long head of the biceps brachii tendon”) and (injury* or torn* or wound* or tear* or “*treatment outcome*”)	(1 or 4) and 2 and (3 or 5)
Web of Science	“*rotator cuff*” and (*biceps* or “long head of the biceps tendon”) and (injury* or torn* or wound* or dislocation* or tear* or “*treatment outcome*”) and “*clinical trial*”	1 and 2 and (3 or 5) and 6
PEDro	“*rotator cuff”* and *biceps*	1 and 2
Scopus	*“rotator cuff”* and (*biceps* or “long head of the biceps tendon”) and (injury* or torn* or wound* or dislocation* or tear* or “*treatment outcome*”) and “*clinical trial”*	1 and 2 and (3 or 5) and 6
Cihnal	“*rotator cuff”* and *biceps* and “*clinical trial”*	1 and 2 and 6
Dialnet	(“manguito de los rotadores” or bíceps) and (lesión* or desgarro* or luxación*)	(7 or 8) and 9

Injury*: injury, injuries; torn*: torn, torns; wound*: wound, wounds; dislocation*: dislocation, dislocations; tear*: tear, tears; lesión*: lesión, lesiones; desgarro*: desgarro, desgarros; luxación*: luxación, luxaciones. *Mesh term printed in italics*

### Study selection and inclusion/exclusion criteria

The selected studies had to meet the following *inclusion criteria*: (1) studies published until April 22, 2021; (2) population: adults with lesions diagnosed for both RC tendons and LHBT, without racial or gender limits; (3) intervention: surgical treatment, whether it had a postsurgical intervention or not, of RC tendons and LHBT pathologies, to achieve one or more of the following objectives: to decrease pain, to increase range of motion (ROM), patient strength and functionality; (4) study design: randomized clinical trials with a minimum score of 6 in the PEDro scale; (5) language: studies reported in English and Spanish.

The *exclusion criteria* were as follows: studies that included subjects with any neurogenic disorder or with tumors that affected the shoulder.

The title and summary of the found articles determined whether they fulfilled the inclusion criteria in a first phase. Subsequently, we reviewed the full text of the pre-selected studies and documented the reason for excluding the discarded records.

### Data extraction

Data extraction was carried out by one reviewer (RA) and verified by a second reviewer (VP). Disagreements between reviewers were resolved by a third reviewer (GC), who assessed the information independently to resolve the discrepancies.

A pre-designed table detailed information on study features, participant characteristics, diagnostic methods, dysfunctions and injury frequency, surgical and postsurgical interventions, outcome measures (functional rating scales like Constant or tools like dynamometers, among others) and results (e.g., pain, ROM, patient strength, and functionality).

The methodological quality data were collected in a standardized table. See the section “Quality appraisal.”

### Quality appraisal

The methodological quality of trials was assessed by using the Physiotherapy Evidence Database (PEDro) scale [13], which evaluates both the internal validity of the study and the adequacy of the statistical information to interpret the results [14]. The scale is composed of 11 items, although the first criterion is not included in the final marker [14], so the maximum score is 10. All PEDro items assess the risk of bias of the selected studies, as shown in the results section of the key for Table 3. They were considered as excellent quality between 9 and 10 points, good quality between 6 and 8 points, fair quality for 4–5 points, and poor methodological quality for below 4 points.

The evidence levels determined by the authors of the included studies will also be considered.

## Results

### Search results

**Fig. 1:**
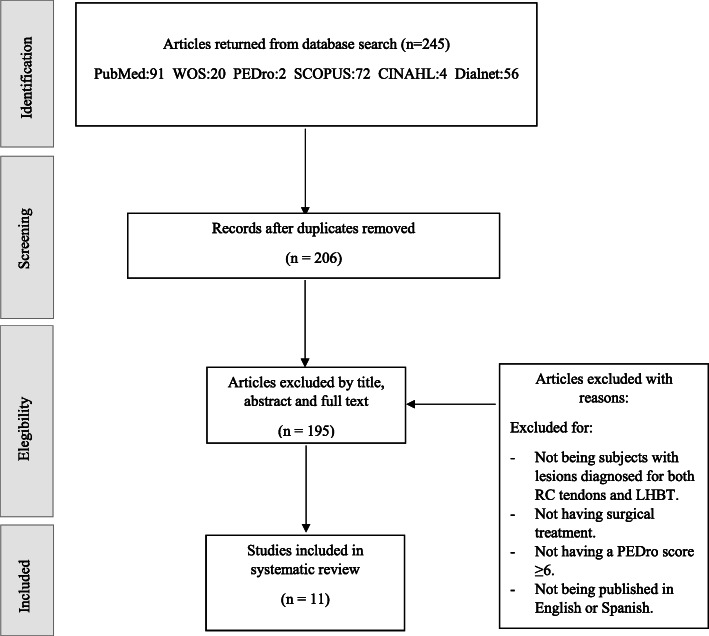
PRISMA flow diagram

The initial search produced 245 results. Following the removal of duplicates, 206 articles were screened by title, abstract, and full-text, due to as follows: not including subjects with lesions diagnosed for both RC tendons and LHBT, not having surgical treatment, not having ≥ 6 score in PEDro scale, not publishing in English or Spanish. Finally, 11 studies [15–25] were included in this review. Figure 1 shows study selection process based on PRISMA [12].

### Characteristics of included studies

A detailed summary of features and results of each selected study is shown in Table 2.

**Table 2:** Characteristics of included studies

Study characteristics	Participant characteristics and diagnostic methods	Dysfunctions and frequency injuries	Interventions	Outcome measures and results
Franceschi et al. [15] To demonstrate that in patients over 50 years of age with arthroscopically confirmed lesions of the RC and a type II SLAP (labrum and LHBT), there is no difference between (1) repair of both lesions and (2) repair of the RC tear without repair of the type II SLAP lesion but with tenotomy of the LHBT. Level of evidence: I (determined by the authors).	*n* = 63. 33 men (52.38%); 30 women (47.62%). Mean age = 63.25. Dominant arm = 76.19%.**Inclusion criteria:** - RC tear diagnosed, - > 50 years, - No episodes of shoulder instability, - No radiographic signs fx of the glenoid or the greater or lesser tuberosity, - MRI evidence of RC tear and type II SLAP lesion, - Symptoms at least 3 months - Inadequate response to conservative management, - RC tear and a type SLAP II lesion found at the time of surgery.**Exclusion criteria:** - Inflammatory joint disease, - Prior surgery on the affected shoulder, an arthroscopic diagnosis of SbT tear, - Inability to complete questionnaires. **Diagnostic: **arthroscopy, MRI and RX.	RC tears: Tear size: • Small (< 1 cm): 30.15%. • Medium (1–3 cm): 36.51%. • Large (3–5 cm): 33.33%. Tear shape: • Crescent shaped: 49.20%. • L-shaped: 28.58%. • U-shaped: 22.22%. Involved tendons: • ST: 58.73%. • ST and IT: 41.27%. Type II SLAP lesion: 100%; • Anterosuperior type II: 34.92%. • Posterosuperior type II: 25.40%. • Combined anterosuperior and posterosuperior type II: 39.68%.	Surgical technique: • EG1: arthroscopy; RC repair and type II SLAP lesion repair (labrum+LHBT). • EG2: arthroscopy; RC repair and biceps tenotomy. Postoperative management: • Sling with an abduction pillow (6 weeks). • Active elbow flexion and extension were allowed, but terminal extension was restricted. Passive ER (1 day after surgery). • Overhead stretching was restricted (6 weeks postoperatively). • At 6 weeks, the sling was removed, and overhead stretching were started (rope and pulley). • Isoinertial strengthening and rehabilitation of RC, deltoid, and scapular stabilizers were initiated (10 or 12 weeks post-operation). • Rehabilitation continued 6 months. • Heavy manual work and overhead activities were allowed after 6 to 10 months after surgery.	The mean follow-up was 5.2 years. **Comparison between pre-intervention and post-intervention** *UCLA rating system(0–35 points):* EG1: 10.4 (range, 6–14) vs 27.9 (range, 24–35); *p*< 0.001. EG2: 10.1 (range, 5–14) vs 32.1 (range, 30–35); *p* < 0.001. *ROM goniometer:* Flexion: EG1: 107° (range 30°–140°) vs 139° (120°–170°); *p* < 0.001. EG2: 99° (range, 30°–140°) vs 166° (range, 140°–170°); *p* < 0.001. ER: EG1: 81.7° (range, 6°–95°) vs 121.4° (range, 90°–140°); *p* < 0.001. EG2: 76.6° (range, 60°–90°) vs 134.3° (range, 90°–140°); *p* < 0.001. IR: EG1: 26.0° (range, 20°–33°) vs 34.3° (range, 26°–40°); *p* < 0.001. EG2: 29.1° (range, 21°–35°) vs 40.0° (range, 30°–45°); *p* < 0.001. **Comparison between the 2 groups postoperatively** There was a statistically significant difference in total postoperative UCLA scores and ROM (*p* < 0.05).
Franceschi et al. [16] To determine whether or not to detach the biceps tendon from the glenoid after tenodesis performed with the inclusion of the biceps in the RC suture results in an improved outcome. Level of evidence: not specified.	*n* = 22. 11 men (50%); 11 women (50%) Mean age = 59.2. Dominant arm = yes. EG1: Dominant/Not dominant: 8/11: 72.7%. EG2: Dominant/Not dominant: 8/11: 72.7%.**Inclusion criteria:** - No improvements after preoperative conservative treatments, - Had at least one positive biceps test.**Exclusion criteria:** - Prior surgical procedure on the affected shoulder. **Diagnostic:** arthroscopy, MRI and RX.	RC tears: Involved tendons: • 3 tendons: 27.27%. • ST and IT: 13.64%. • ST and SbT: 18.18%. • ST: 40.91%. LHBT: • Tear > 50%: 36.36%. • Dislocated: 31.82%. • Unstable: 31.82%.	Surgical technique: • EG1: arthroscopy; RC repair and tenodesis without tenotomy of LHBT. • EG2: arthroscopy; RC repair and tenodesis with tenotomy of LHBT. Postoperative management: • Sling with an abduction pillow (6 weeks). • Active elbow flexion and extension were allowed, but terminal extension was restricted. • Passive ER (1 day after surgery). • Overhead stretching was restricted (6 weeks postoperatively). • At 6 weeks, the sling was removed, and overhead stretching were started (rope and pulley). • Isoinertial strengthening and rehabilitation of RC, deltoid, and scapular stabilizers were initiated (10 or 12 weeks post-operation). • Rehabilitation continued 6 months. • Heavy manual work and overhead activities were allowed after 6 to 10 months after surgery.	The mean follow-up was 47.2 months. **Comparison between pre-intervention and post-intervention** *UCLA Score*0–35 points):(*0–35 points):* EG1: 10.5 vs 33; *p* < 0.05. EG2: 11.1 vs 32.9; *p* < 0.05. *ROM goniometer:* Flexion: EG1: 102° (range 30°–140°) vs 161° (range 150°–170°); *p* < 0.05. EG2: 110° (range 30°–150°) vs 159° (range 140°–170°); *p* < 0.05. ER: EG1: 37° (range 30°–60°) vs 59° (range 45°–70°); *p* < 0.05. EG2: 41° (range 30°–60°) vs 60° (range 45°–90°); *p* < 0.05. IR: EG1: (L5 a T10) vs (T11 a T5). EG2: (L5 a T12) vs (T12 a T5). **Comparison between groups** did not show statistically significant differences.
Grasso et al. [17] To compare the clinical outcome of arthroscopic RC repair with single-row and double-row techniques. Level of evidence: I (determined by the authors).	*n* = 72. 34 men (47.22%) 38 women (52.77%). Mean age = 56.8. Dominant arm = yes. Dominant = 77.77%.**Inclusion criteria:** -Repairable full-thickness tear of the supraspinatus or the posterior superior RC, with rotator interval involvement or biceps pathology.**Exclusion criteria:** - Partial-thickness or irreparable full-thickness tear, subscapularis tendon tear, an isolated subscapularis tear, labral pathology amenable to surgical repair, -Degenerative arthritis glenohumeral joint, - Symptomatic arthritis of the acromioclavicular joint, - RC arthropathy, - Previous surgery on the same shoulder, - Workers’ Compensation claims. **Diagnostic:** arthroscopy and MRI.	RC tears: Involved tendons: • ST: 54.17%. • ST and part of IT: 26.38%. • ST and IT: 19.44%. Tear shape: • Crescent shaped: 48.61%. • L-shaped: 15.28%. • Inverse L shaped: 13.89%. • V-shaped: 19.44%. • U-shaped: 2.78%. LHB: biceps pathology.	Surgical Technique: • EG1: arthroscopy; RC repair with single-row and biceps tenodesis or tenotomy (depending on the patient’s age: > 50, tenotomy). • EG2: arthroscopy; RC repair with double-row and biceps tenodesis or tenotomy. Postoperative rehabilitation: A sling during 3 weeks; after this, the following rehabilitation program: • 1 phase (4–8 weeks after surgery): ROM exercise program (passive, active assisted, and active). • 2 phase (9–12 weeks after surgery): Muscle-strengthening program using closed kinetic chain exercises for RC, subscapularis, biceps, deltoid, pectoralis major, and scapular stabilizers. • 3 phase (13–16 weeks after surgery): Open kinetic chain exercises, proprioceptive and plyometric exercises, and postural rehabilitation of kinetic chain (lumbopelvic, thoracolumbar, and scapulothoracic regions).	The mean follow-up was 24.8 ± 1.4 months. **Comparison between groups** did not show significant differences. *DASH score* *(0–100 points):* EG1 (15.4 ± 15.6) vs EG2 (12.7 ± 10.1); *p* = 0.482. Work-DASH score *(0–100 points):* EG1 (16.0 ± 22.0) vs EG2 (9.6 ± 13.3); *p* = 0.212. * Constant Score (0–100 points):* EG1 (100.5 ± 17.8) vs EG2 (104.9 ± 21.8); *p* = 0.378. Muscle strength evaluation with a digital *dynamometer:* EG1 (12.7 ± 5.7) vs EG2 (12.9 ± 7.0); *p* = 0.382.
Lee et al. [18] To compare the clinical outcomes of tenotomy with those of tenodesis for treatment of LHBT lesions in patients with RC tears. Level of evidence: I (determined by the authors).	*n* = 128. 29 men (22.65%); 99 women (77.34%). Mean age = 62.85. Dominant arm = no. **Inclusion criteria:** - Symptomatic LHBT partial tears and small- to medium-sized RC tears, - No improvements after conservative treatments (1 month). **Exclusion criteria:** - Large or massive RC tears - History of shoulder surgery or trauma or concomitant shoulder lesions. **Diagnostic:** arthroscopy and MRI.	RC tears: 100%. Size: small (< 1 cm) or medium (1–3 cm). LHBT: • LHBT tears: 65.62%. • Subluxated: 23.44%. • Dislocated: 10.94%.	Surgical technique: • EG1: arthroscopy; RC repair (single row for small RC tears and transosseous equivalent repair for medium RC tears) and LHBT tenotomy. • EG2: arthroscopy; RC repair and LHBT tenotomy with tenodesis. Postoperative rehabilitation: • Abduction brace immediately after the operation for 4 weeks postoperatively. • Afterwards, pulley exercises were prescribed to increase their range of flexion. • Home-based active assisted shoulder exercises; 3 times daily, each session 20 min. • Elastic band exercises, strengthening exercises for the muscles stabilizing the scapula, were initiated 2 months after the operation. • All sports activities were permitted after 6 months.	The mean follow-up was 25.1 months in EG1 and 19.7 months in EG2. **Comparison between pre-intervention and post-intervention** *Pain (VAS0–10)*: EG1: (7.1 vs 2.0); *p* = 0.02. EG2: (5.9 vs 1.8); *p* = 0.03. *ASES score (0–100 points)*: EG1: (44.2 vs 82.8); *p* = 0.01. EG2: (51.5 vs 77.6); *p* = 0.02. *Constant Score (0-100 points): ****(0–100 points)*** EG1: (69.9 vs 88.3); *p* = 0.03. EG2: (69.9 vs 86.5); *p* = 0.02. **Comparison between groups** 12 months post-operatively: no significant differences were found in ROM, pain and functional scores between the groups (*p* > 0.05). *Popeye´s deformity* was 3 times higher in group I than in group II (19.6% vs 5.6%) *p* = 0.04. Besides, group II showed *greater forearm supination* power (0.818 ± 0.108) than group I (0.998 ± 0.015, *p* = 0.02).
Zhang et al. [19] To compare the clinical outcomes between tenotomy and tenodesis for the treatment of LHB lesions in patients > 55 years of age affected by reparable RC tears with concomitant LHB pathologies. Level of evidence: I (determined by the authors).	*n* = 151. 71 men (47.02%) 80 women (52.98%). Mean age = 61. Dominant arm = no. **Inclusion criteria:** Inclusion criteria: - Had not improved after non-operative treatments, -Affected by both RC tears and LHBT pathologies.**Exclusion criteria:** Exclusion criteria: - < 55 years, - Previous surgical treatment on the affected shoulder, - Radiological signs of glenohumeral arthritis, and disability at the contralateral arm. -Small to large full-thickness RC tears and massive irreparable tears. **Diagnostic: **Physical examination, arthroscopy, MRI, US and RX.	RC tears: Size: • Small: 47.01%. • Medium: 33.77%. • Large: 19.22%. Biceps tendon pathologies (some cases overlapped): • Severe inflammation: 26.49%. • Tears more than 25 %: 68.21%. • Subluxations or dislocations: 20.53%. • Type II or type IV SLAP lesions: 23.84%.	Surgical technique: • EG1: arthroscopy; RC repair and LHBT tenotomy. • EG2: arthroscopy; RC repair and LHBT tenotomy with tenodesis. Postoperative rehabilitation: • All the patients followed the routine rehabilitation procedures after RC repair. • For tenotomy group: immobilization of their elbow motion for 1 week. • Active ROM and gentle strength training 6 weeks post operation. • Unrestricted use of the biceps muscle was not allowed until 16–20 weeks post operation.	Follow-up average of 25 months. **Comparison between groups** *Constant Score (0–100 points):* EG1 (95.6 ± 3.0) vs EG2 (96.5 ± 2.6); NS. *Muscle strength measured with a digital dynamometer:* Flexion: EG1 (0.9 ± 0.2) vs EG2 (0.9 ± 0.2); NS. Supination: EG1 (0.9 ± 0.2) vs EG2 (0.9 ± 0.1; NS.*Popeye´s sign:* EG1 (7) vs EG2 (2); NS. *Pain* (VAS, 4 weeks post-operatively): EG1 (2.0 ± 1.1) vs EG2 (2.1 ± 1.6); NS.*Degree of satisfaction:* Excellent or good: EG1 (65) vs EG2 (60); NS. Fair: EG1 (12) vs EG2 (13); NS. Poor: EG1 (0) vs EG2 (1); NS. Tenotomy (EG1) required a shorter *surgical time* (40.4 ± 4.0 vs. 50.4 ± 5.9 min; *p* < 0.001) and resulted in faster pain relief, 2 weeks post-operatively (3.1 ± 1.8 vs. 4.8 ± 1.9; *p* < 0.001).
Park et al. [20] To compare the clinical and anatomic outcomes of the interference screw and suture anchor fixation techniques for biceps tenodesis performed along with arthroscopic RC repair. Level of evidence: II (determined by the authors).	*n* = 67. 28 men (41.79%); 39 women (58.21%). Mean age = 61.8 Dominant arm = no.**Inclusion criteria:** - Partial or full-thickness RC tears by preoperative MR arthrography. - Concomitant biceps lesions (LHBT partial tear > 50%, type II SLAP lesion, pulley lesion, or subluxation/dislocation of the LHBT). -Arthroscopic RC repair.**Exclusion criteria:** - Isolated glenohumeral pathological conditions (e.g., SLAP lesion or instability), - Previous surgery on the same shoulder, - Complete ruptures of the LHBT, - Incomplete repair of the RC, - Symptomatic acromioclavicular arthritis, - Refusal to be enrolled. **Diagnostic:** arthroscopy, MRI and RX.	RC tears: 100%. Biceps tendon pathologies (some cases overlapped): EG1: Biceps tears: 48.48%. SLAP lesion: 63.63%. Pulley lesion: 39.39%. Subluxation: 24.24%. Dislocation: 15.15%. EG2: Biceps tears: 52.94%. SLAP lesion: 55.88%. Pulley lesion: 29.41%. Subluxation: 8.82%. Dislocation: 20.58%.	Surgical technique: • EG1: arthroscopy; RC repair and screw fixation techniques for biceps tenodesis. • EG2: arthroscopy; RC repair and suture anchor fixation techniques for biceps tenodesis. Postoperative rehabilitation: • Immobilization in abduction: 4 weeks for a partial-thickness and small (< 1 cm) tear, 5 weeks for a medium (1–3 cm) tear, and 6 weeks for a large to massive tear (> 3 cm). • Shrugging of both shoulders, active elbow flexion/extension, active forearm supination/pronation, and active hand and wrist motion were encouraged immediately after surgery. • Active-assisted shoulder ROM exercises were encouraged after weaning from the brace. • Muscle strengthening exercises were started at 9 to 12 weeks postoperatively. • All sports activities were permitted from 6 months after surgery.	The mean follow-up 27.7 ± 6.41 months 28.8 ± 7.3 months in the EG1 group 26.6 ± 5.3 months in the EG2. **Comparison between pre-intervention and post-intervention** *Pain* (VAS, 0–10): (6.7 ± 2.0) vs (0.6 ± 1.0); *p* < 0.001. ASES score *ASES score *(0–100 points): (53.7 ± 16.5) vs (93.2 ± 9.5); *p* < 0.001. *KSS *(0–100 points): (50.5 ± 11.7) vs (94.4 ± 6.2); *p* < 0.001. Constant Score (0–100 points): (51.8 ± 9.3) vs (69.6 ± 7.7); *p* < 0.001. SST score(0–12 points): (4.7 ± 2.1) vs (10.4 ± 2.4); *p* < 0.001.*Flexion;* (158.7 ± 13.6) vs (167.2 ± 22.7); *p* = 0.007. * ER:* (60.5 ± 12.1) vs (70.8 ± 17.1); *p* < 0.001.*IR:* (9.4 ± 2.4) vs (8.3 ± 1.8); *p* = 0.007. **Comparison between groups** *Pain:* (VAS, 0–10): EG1 (0.5 ± 0.9) vs EG2 (0.8 ± 1.2); *p* = 0.225. *ASES *(0–100 points): EG1 (93.9 ± 9.2) vs EG2 (92.4 ± 9.8); *p* = 0.507. *KSS* (0–100 points): EG1 (94.9 ± 6.1) vs EG2 (93.8 ± 6.3); *p* = 0.487. *Constant* (0–100 points): EG1 (69.3 ± 8.3) vs EG2 (69.9 ± 7.2); *p* = 0.720. *SST score *(0–12 points): EG1 (8.9 ± 1.2) vs EG2 (8.8 ± 1.1); *p* = 0.595.*Flexion:* EG1 (170.1 ± 12.5) vs EG2 (164.5 ± 29.4); *p* = 0.327.*ER:* EG1 (72.1 ± 20.5) vs EG2 (70.1 ± 13.2); *p* = 0.643.*IR:* EG1 (8.6 ± 1.8) vs EG2 (8.1 ± 1.8); *p* = 0.219.*Popeye´s deformity by patient:* EG1 (35.44) vs EG2 (32.60); *p* = 0.314.*Popeye´s deformity by examiner:* EG1 (34.59) vs EG2 (33.43); *p* = 0.756.
De Carli et al. [21] To determine clinical, functional, and radiological results of two groups of patients affected by RC tears with concomitant degeneration of LHBT treated with tenotomy and tenodesis or tenotomy. Level of evidence: II (determined by the authors).	*n* = 65. 48 men (74%); 17 women (26 %). Mean age = 57.95. Dominant arm = no. **Inclusion criteria:** - Diagnosis of a small to large RC tear and the presence of an associated degenerative lesion of the LHB (including degenerative tears, tenosynovitis, subluxation on the medial rim of the bicipital groove, and SLAP lesions). **Exclusion criteria:** - Previous surgical treatment on shoulder, - > 65 years, - Radiological signs of glenohumeral arthritis, - Grade 3 or 4 degeneration according to Goutallier. **Diagnostic:** MRI, RX and arthroscopy.	RC tears: 100%. Biceps pathology: EG1: Biceps tears: 65%. Tenosynovitis: 20%. Degenerative SLAP lesion: 15%. EG2: Biceps tears: 67%. Tenosynovitis: 23%. Degenerative SLAP lesion: 10%.	Surgical technique: • EG1: arthroscopy; RC repair and LHBT tenotomy with tenodesis. • EG2: arthroscopy; RC repair and LHBT tenotomy.	The mean follow-up was 24 months. **Comparison between groups** No significant differences between the groups: *Constant Score* (0–100 points): Pre-treatment: EG1 (44.1 ± 6.3) vs EG2 (47.4 ± 12.1); NS. Post-treatment: EG1 (97.2 ± 4.9) vs EG2 (94.6 ± 4.9); NS. *SST* (0–12 points): Pre-treatment: EG1 (4.4 ± 0.8) vs EG2 (4.4 ± 0.7); NS. Post-treatment: EG1 (11.7 ± 1.3) vs EG2 (10.6 ± 1.3); NS. *Muscle strength measured with a digital dynamometer* (dynamometric test): This test compared the operated side with health side. Flexion shoulder (operated side vs health side): EG1: (9.8 ± 4) vs (10.8 ± 3.5); *p* = 0.02. EG2: (9.8 ± 2.4) vs (10.6 ± 2.4); *p* = 0.02. Extension shoulder abducated: EG1: (12.1 ± 4) vs (13.3 ± 2.9); *p* = 0.01. EG2: (1.7 ± 2.4) vs (12.9 ± 17); *p* = 0.01. Flexion forearm shoulder abducated: EG1: (7.5 ± 2.5) vs (8.7 ± 2.7); *p* = 0.01. EG2: (7.6 ± 3.1) vs (8.8 ± 1.6); *p* = 0.02. Abduction shoulder abducated 45°: EG1: (7.2 ± 1) vs (9 ± 4.2); *p* = 0.001. EG2: (6.8 ± 1.1) vs (8 ± 0.8); *p* = 0.001.*Popeye´s deformity:* A significant in 17% of patients treated with tenotomy (*p* < 0.05).
Castricini et al. [22] To compare the effectiveness of tenodesis and tenotomy in the treatment of LHBT lesions. Level of evidence: I (determined by the authors).	*n* = 55. 21 men (38.18%) 34 women (61.82 %). Mean age = 58.5. Dominant arm = yes. Dominant = 81.82% .**Inclusion criteria:** - Grade I or II full-thickness reparable supraspinatus tendon tear with a LHBT lesion, - > 40 years. **Exclusion criteria:** - Prior surgery on the affected shoulder, a lack of willingness to return for all scheduled follow-up visits, - Previous upper extremity neurological disorder or diagnosis based upon physical examination, - Complaint of pain in both shoulders, - Life expectancy < 2 years - Insurance trial, lawsuit, or pending legal action for shoulder disease. **Diagnostic:** MRI and RX.	RC tears: ST tears 100%. Biceps pathology: EG1: Biceps tendon instability: Stable: 48.4%. Unstable: 51.6%. Biceps tendon lesion (partial ruptured of the tendon): Normal: 71%. Minor lesion: 3.2%. Major lesion: 25.8% EG2: Biceps tendon instability: Stable: 20.8%. Unstable: 79.2%. Biceps tendon lesion (partial ruptured of the tendon): Normal: 87.5%. Minor lesion: 8.3%. Major lesion: 4.2%	Surgical technique: • EG1: arthroscopy; RC repair and LHBT tenotomy. • EG2: arthroscopy; RC repair and LHBT tenotomy and tenodesis. Postoperative rehabilitation: • Immobilization was maintained with a 20° abduction pillow for 3 weeks. • Pendulum exercises were allowed, starting from the first post-operative day. • After the immobilization period, passive and assisted exercises in forward flexion and external rotation were initiated. • Strengthening exercises were restricted until 6 weeks after the surgical procedure. • 3 months after the operation, patients were allowed to engage in light physical sports activity. • Heavy manual work and overhead motion were allowed after 6 months.	The follow-up was 6 and 24 months. **Comparison between groups** No significant differences between the groups *Constant Score *(0–100 points): Pre-treatment: EG1 (48.1 ± 4.7) vs EG2 (47 ± 6.3); NS. Post-treatment: 6 months EG1 (75.1 ± 8.1) vs EG2 (75 ± 8.1); NS. Post- treatment: 24 months EG1 (85.2 ± 8.1) vs EG2 (84.4 ± 6.5); NS. *Pain* (VAS, 0–10): Post-treatment: 6 months EG1 (1.1 ± 1.9) vs EG2 (1.5 ± 2); NS. Post-treatment: 24 months EG1 (1 ± 1.9) vs EG2 (1 ± 2); NS. *Muscle strength measured with a digital dynamometer* (dynamometric test): Post-treatment: 6 months Abduction: EG1 (1.8 ± 1) vs EG2 (1.8 ± 1.4); NS. Elbow flexion: EG1 (12.1 ± 7) vs EG2 (10.5 ± 8.1); NS. External rotation: EG1 (5.9 ± 2.8) vs EG2 (5 ± 3.7); NS. Post-treatment: 24 months Abduction: EG1 (6 ± 2.6) vs EG2 (5.2 ± 2.6); NS. Elbow flexion: EG1 (14.6 ± 8.8) vs EG2 (11.1 ± 6.4); NS. External rotation: EG1 (8.6 ± 4.8) vs EG2 (6.2 ± 3.7); NS. *SF-36 Health Survey* (0–100 points): Pre-treatment: Physical Component Summary: EG1 (57.4 ± 20) vs EG2 (60.2 ± 24.4); NS. Mental Component Summary: EG1 (61.3 ± 16.3) vs EG2 (62.2 ± 22.7); NS. Post-treatment: 6 months Physical Component Summary: EG1 (49 ± 10.1) vs EG2 (49.8 ± 10.2); NS. Mental Component Summary: EG1 (51.7 ± 7.5) vs EG2 (50 ± 8.2); NS. Post- treatment: 24 months Physical Component Summary: EG1 (52.4 ± 6.8) vs EG2 (51.6 ± 6.5); NS. Mental Component Summary: EG1 (49.9 ± 12.8) vs EG2 (51.2 ± 6.4); NS. *Biceps cramping:* Post-treatment: 6 months EG1 (0) vs EG2 (3); *p* = 0.043. Post- treatment: 24 months No cases were noted in both groups.*Popeye´s sign* Post-treatment: 6 months EG1 (17) vs EG2 (2); *p* < 0.001. Post-treatment: 24 months EG1 (18) vs EG2 (5); *p* = 0.006.
Mardani-Kivi et al. [23] To evaluate outcomes of tenotomy and tenodesis in the treatment of LHBT lesions with RC tears and to compare their advantages and disadvantages. Level of evidence: II (determined by the authors).	*n* = 62. 42 men (67.7%) 20 women (32.3%). Mean age = 55. Dominant arm = yes. Dominant = 56.5%. **Inclusion criteria:** - Patients aged 45 to 60 years. - Candidates for arthroscopic healing of RC tears. - At least 1 positive biceps test before surgery and who had inflammation, partial tears, or luxation or SLAP lesions during surgery .**Exclusion criteria:** - Patients with extensive fatty infiltration between ruptured RC on MRI, - Positive history of steroid injection or physical therapy, - Popeye’s deformity, tumors or cysts in the area of the bicipital groove and the proximal humeral shaft, - Pain in both shoulders, - Impossibility of arthroscopic RC repair during surgery, and conversion to open surgery. **Diagnostic:** MRI, arthroscopy and biceps test.	RC tears: Size: EG1: • Small: 34.5%. • Medium: 27.6%. • Large: 24.1%. • Massive: 13.8%. • EG2: • Small: 36.4%. • Medium: 24.2%. • Large: 24.2 %. Massive: 15.2%. Biceps tendon pathologies: EG1: Tendinosis: 44.8%. SLAP lesion: 10.3%. Partial tear: 31%. Instability: 13.8%. EG2: Tendinosis: 42.4%. SLAP lesion: 12.1%. Partial tear: 33.3%. Instability: 12.1%.	Surgical technique: • EG1: arthroscopy; RC repair and tenotomy of LHBT. • EG2: arthroscopy; RC repair and tenotomy + open surgery; subpectoral tenodesis of LHBT. Postoperative rehabilitation: • The first 6 weeks after operation, sling and abduction pad were used. Elbow extension and flexion were allowed, but terminal extension was forbidden. • 1st postoperative day: passive external rotation was started. • Pull over was forbidden up to 6 weeks to prevent damage of the healing area. • After 6 weeks, the sling was removed and pull over was started with the help of tackles. • Isotonic strengthening of fixator muscles of RC, deltoid, and scapula were started at 10th to 12th postoperative weeks. • This rehabilitation process was continued for 6 months. • Heavy hand work and pull over activities were allowed after 6 to 10 postoperative months.	The follow-up was 6, 12 and 24 months. **Comparison between preoperative and postoperative treatment** **EG1: (before) vs (6 months post-treatment)** *Constant* (0–100 points): (61.01 ± 6.12) vs (73.07 ± 5.85); *p* < 0.001. *Pain* (NRS, 0–100 points): (77.88 ± 12.27) vs (46.27 ± 7.25); *p* < 0.001. *SST* (0–12 points): (4.07 ± 1.66) vs (7.34 ± 1.34); *p* < 0.001. *Patient satisfaction* (VAS, 0–10): (1.96 ± 1.22) vs (6.38 ± 0.60); *p* < 0.001. **EG1: (before) vs (12 months post-treatment)** Constant (0–100 points): (61.01 ± 6.12) vs (82.14 ± 7.93); *p* < 0.001. Pain (NRS, 0–100 points): (77.88 ± 12.27) vs (1.70 ± 3.07); *p* < 0.001. SST (0–12 points): (4.07 ± 1.66) vs (9.17 ± 1.44); *p* < 0.001. Patient satisfaction (VAS, 0–10): (1.96 ± 1.22) vs (8.07 ± 0.66); *p* < 0.001. **EG1: (before) vs (24 months post- treatment)** *Constant* (0–100 points): (61.01 ± 6.12) vs (88.1 ± 5.4); *p* < 0.001. *Pain* NRS, 0–100 points): (77.88 ± 12.27) vs (0.35 ± 0.85); *p* < 0.001. *SST *(0–12 points): (4.07 ± 1.66) vs (11.14 ± 0.74); *p* < 0.001. *Patient satisfaction* (VAS, 0–10): (1.96 ± 1.22) vs (9.07 ± 0.58); *p* < 0.001. **EG2: (before) vs (6 months post-treatment)** *Constant* (0–100 points): (61.76 ± 8.07) vs (73.12 ± 6.83); *p* < 0.001. *Pain* (NRS, 0–100 points): (79.57 ± 11.80) vs (46.88 ± 5.61); *p* < 0.001. *SST* (0–12 points): (4 ± 1.27) vs (7.40 ± 1.65); *p* < 0.001. *Patient satisfaction* (VAS, 0–10): (2.01 ± 1.23) vs (6.10 ± 0.74); *p* < 0.001. **EG2: (before) vs (12 months post-treatment)** *Cosntant* (0–100 points): (61.76 ± 8.07) vs (83.51 ± 5.13); *p* < 0.001. *Pain* (NRS, 0–100 points): (79.57 ± 11.80) vs (2.27 ± 4.04); *p* < 0.001. *SST* (0–12 points): (4 ± 1.27) vs (8.70 ± 1.51); *p* < 0.001. *Patient satisfaction* (VAS, 0–10): (2.01 ± 1.23) vs (8.61 ± 0.66); *p* < 0.001. **EG2: (before) vs (24 months post-treatment)** *Constant *(0–100 points): (61.76 ± 8.07) vs (89.94 ± 3.24); *p* < 0.001. *Pain* (NRS, 0–100 points): (79.57 ± 11.80) vs (0.48 ± 1.27); *p* < 0.001. *SST* (0–12 points): (4 ± 1.27) vs (11.42 ± 0.87); *p* < 0.001. *Patient satisfaction* (VAS, 0–10): (2.01 ± 1.23) vs (9.53 ± 0.48); *p* < 0.001. **Comparison between EG1 vs EG2**: 12 months post- treatment: *Patient satisfaction* (VAS, 0–10): EG1 (8.07 ± 0.66) vs EG2 (8.61 ± 0.66); *p* = 0.003. 24 months post-treatment:24 months post-treatment: *Patient satisfaction* (VAS, 0–10): EG1 (9.07 ± 0.58) vs EG2 (9.53 ± 0.48); *p* = 0.001. *Popeye´s sign:* EG1 (7) vs EG2 (1); *p* = 0.017.*Cramping:* EG1 (9) vs EG2 (0); *p* = 0.0001.
Mardani-Kivi et al. [24] To compare clinical and functional outcomes of open subpectoral versus arthroscopic intraarticular tenodesis in patients with reparable RC tear associated with LHBT degeneration. Level of evidence: II (determined by the authors).	*n* = 60. 26 men (43.3%) 34 women (56.6%). Mean age = 55.7 Dominant arm = yes. Dominant = 81.6%. **Inclusion criteria:** - Age 18 to 65 years, - Candidates for arthroscopic repair RC tear with anterior shoulder pain, - At least 1 positive biceps test and who also had subluxation, dislocation, partial tear or SLAP lesion on arthroscopic evaluations. - No evidence of extensive fatty infiltration in ruptured RC muscles on MRI. **Exclusion criteria**: - Previous shoulder surgery, -Tumors or cysts in the area of the bicipital groove and the proximal humeral shaft, - Pain in both shoulders, - Impossibility of arthroscopic RC tear repair during surgery and conversion to open surgery. **Diagnostic:** MRI, arthroscopy and biceps test.	RC tears: 100%. Biceps tendon pathologies: subluxation, dislocation, partial tear or SLAP lesion.	Surgical technique: • EG1: arthroscopy; RC repair and tenodesis of LHBT. • EG2: arthroscopy; RC repair + open surgery; subpectoral tenodesis of LHBT. Postoperative rehabilitation: • The first 6 weeks after surgery, a sling with abduction pad was used. • Active flexion and extension of the elbow were allowed, but terminal extension was forbidden. • After 6 weeks, the sling was removed. • Isotonic strengthening of the fixator muscles of RC, deltoid, and scapula was started at 10 to 12 postoperative weeks. • This rehabilitation process was continued for 6 months. • Heavy manual work and overhead activities were allowed only after sufficient muscle strengthening at approximately 6–10 months after surgery.	The follow-up was 6 and 24 months. **Comparison between EG1 vs EG2 (6 month follow-up):** *Constant Score *(0–100 points): EG1 (82.1 ± 5.6) vs EG2 (81.2 ± 6.9); NS. *SST *(0–12 points): EG1 (10.2 ± 0.7) vs EG2 (10.2 ± 0.8); NS. *Pain* (VAS, 0–10): EG1 (2 ± 0.8) vs EG2 (2.2 ± 0.9); NS. **Comparison between EG1 vs EG2 (2 year follow-up):** *Constant Score *(0–100 points): EG1 (93.1 ± 3.9) vs EG2 (92.7 ± 5.2); NS. *SST *(0–12 points): EG1 (11.5 ± 0.7) vs EG2 (11.3 ± 0.8); NS. *Pain* (VAS, 0–10): EG1 (0.4 ± 0.6) vs EG2 (0.4 ± 0.5); NS. *Patient satisfaction* (VAS, 0–10) between EG1 and EG2 (9.7 ± 0.5 and 9.5 ± 0.7); *p* = 0.47. Not significantly difference between the two groups.
Van Deurzen et al. [25] To determine if LHB tenotomy is not inferior to suprapectoral LHB tenodesis when performed in conjunction with arthroscopic repair of small-to medium-sized nontraumatic RC tears. Level of evidence: I (determined by the authors).	*n* = 100. 61 men (61%) 39 women (39%). Mean age = 61. Dominant arm = yes. Dominant = 60%. **Inclusion Criteria:** - Patients older than 50 years, - With a nontraumatic small-to medium-sized supraspinatus and/or infraspinatus lesions. - Inflamed or unstable LHBT or an LHB tear greater than 30% encountered during arthroscopic RC repair. **Exclusion criteria:** - Case of a traumatic or partial-thickness RC rupture, full-thickness tear larger than 3 cm, accompanying SbT tear, hourglass deformity or less than 30% tearing of the LHB, SLAP lesions, arthropathy of the glenohumeral joint, acromion-to-humeral head distance measuring 6 mm or smaller, Hamada classification of grade 2 or higher, - Prior surgery on the involved shoulder - Inability to complete the questionnaires and assessments. **Diagnostic:** MRI and arthroscopy.	RC tears: small to medium sized supraspinatus and/or infraspinatus lesions. Biceps tendon pathologies: inflamed or unstable LHBT, LHB tear greater than 30%.	Surgical technique: • EG1: arthroscopy; RC repair (single or double-row technique) and tenotomy of LHBT. • EG2: arthroscopy; RC repair (single or double-row technique) and LHBT tenotomy and tenodesis. Postoperative rehabilitation: • The first 6 weeks after surgery, an immobilizer was used and only passive ROM exercises of the shoulder and elbow were allowed. • After 6 weeks, active movements of both the shoulder and elbow were started and gradually increased. • Full-weight loading of the RC and biceps was not allowed until at least 3 months after surgery.	The mean follow-up period was 12.1 in EG1 and 12.5 months in EG2. **Comparison between preoperative and 1 year postoperative treatment** *Constant Score* (0–100 points) EG1: 44 (95% CI, range 39–48) to 73 (95% CI, range 68–79). EG2: 42 (95% CI, range 37–48) to 78 (95% CI, range 74–82). **Comparison between groups (1 year post-treatment)**: *Constant Score* (0–100 points): EG1 (73.4) vs EG2 (78.2); *p* > 0.06 *Popeye´s sign:* EG1 (47%) vs EG2 (33%); *p* = 0.017. *Patient satisfaction* (5-point Smiley Scale): EG1 vs EG2; *p* = 0.8. The total surgical time was significantly shorter for the EG1 (mean, 73 min) vs EG2 (mean, 82 min): *p* = 0.03.

Note: type II SLAP lesion, detachment of superior labrum and biceps tendon from glenoid rim; type IV SLAP lesion, extension of displaced bucket-handle labral tear into biceps tendon [26]. Values are expressed as mean ± standard deviation unless otherwise stated

Abbreviations: *ASES*, American Shoulder and Elbow Surgeon; *CI*, confidence interval; *DASH*, Disabilities of Arm, Shoulder and Hand; *EG*, experimental group; *ER*, external rotation; *fx*, fracture; *H*, hypothesis; *IR*, internal rotation; *IT*, infraspinatus tendon; *KSS*, Korean Shoulder Scoring system; *LHB*, long head of biceps; *LHBT*, long head of biceps tendon; *MRI*, magnetic resonance imaging; *NRS*, numerical rating scale; *NS*, not significant; RC, rotator cuff; *ROM*, range of motion; *RX*, radiography; *SbT*, subscapularis tendon; *SLAP*, superior labrum anterior to posterior; *SST*, Simple Shoulder Test; *ST*, supraspinatus tendon; *US*, ultrasound; *UCLA*, University of California at Los Angeles Shoulder Score; *VAS*, visual analog scale

### Quality assessment

Table 3 includes the results of the PEDro scale. Ten studies [15–21, 23–25] (90.9%) were considered to be of good methodological quality (6-8 points) and one [22] (9.1%) as excellent quality (9 points).

**Table 3:** Completed PEDro quality appraisal

Studies	Criteria											Total scores
	1	2	3	4	5	6	7	8	9	10	11	
Franceschi et al. [15]	✓	✓	Χ	Χ	Χ	Χ	✓	✓	✓	✓	✓	6
Franceschi et al. [16]	✓	✓	Χ	✓	Χ	Χ	Χ	✓	✓	✓	✓	6
Grasso et al. [17]	✓	✓	✓	✓	Χ	Χ	Χ	✓	✓	✓	✓	7
Lee et al. [18]	✓	✓	Χ	✓	✓	Χ	Χ	✓	✓	✓	✓	7
Zhang et al. [19]	✓	✓	✓	✓	Χ	Χ	✓	✓	✓	✓	✓	8
Park et al. [20]	✓	✓	✓	Χ	✓	Χ	Χ	✓	✓	✓	✓	7
De Carli et al. [21]	✓	✓	Χ	✓	Χ	Χ	Χ	✓	✓	✓	✓	6
Castricini et al. [22]	✓	✓	✓	✓	✓	Χ	✓	✓	✓	✓	✓	9
Mardani-Kivi et al. [23]	✓	✓	Χ	✓	Χ	Χ	Χ	✓	✓	✓	✓	6
Mardani-Kivi et al. [24]	✓	✓	Χ	✓	Χ	Χ	Χ	✓	✓	✓	✓	6
Van Deurzen et al. [25]	✓	✓	✓	✓	✓	Χ	Χ	✓	✓	✓	✓	8

Criteria: (1) Eligibility criteria were specified. (2) Subjects were randomly allocated to groups (in a crossover study, subjects were randomly allocated an order in which treatments were received). (3) Allocation was concealed. (4) The groups were similar at baseline regarding the most important prognostic indicators. (5) There was blinding of all subjects. (6) There was blinding of all therapists who administered the therapy 7. There was blinding of all assessors who measured at least one key outcome. (8) Measures of at least one key outcome were obtained from more than 85% of the subjects initially allocated to groups. (9) All subjects for whom outcome measures were available received the treatment or control condition as allocated or, where this was not the case, data for at least one key outcome was analyzed by “intention to treat.” (10) The results of between-group statistical comparisons are reported for at least one key outcome. (11) The study provides both point measures and measures of variability for at least one key outcome

The items: “subjects were randomly allocated to groups (in a crossover study, subjects were randomly allocated an order in which treatments were received)” [2]; “measures of at least one key outcome were obtained from more than 85% of the subjects initially allocated to groups” [8]; “all subjects for whom outcome measures were available received the treatment or control condition as allocated or, where this was not the case, data for at least one key outcome was analyzed by intention to treat” [9]; “the results of between-group statistical comparisons are reported for at least one key outcome” [10]; and “the study provides both point measures and measures of variability for at least one key outcome” [11], stood out as they were carried out in every study [15–25]. By contrast, item “there was blinding of all therapists who administered the therapy” [6], did not score.

In relation to the evidence levels determined by the authors of the included studies: 6 [15, 17–19, 22, 25] (54.5%) were classified in evidence level I, 4 [20, 21, 23, 24] (36.4%) in level II and 1 [16] (9,1%) was not specified.

### Participant characteristics

The mean sample size of the papers was 77 patients, the smallest being *n* = 22 [16] while the highest was *n* = 151 [19].

In relation to gender, the male sample represents 48% of the total, ranging from 11 [16] to 71 [19] men (mean = 37) compared to 52% of females, between 11 [16] and 99 [18] women (mean = 40).

As for the subjects’ age, the mean was 59.37 years, the one performed by Mardani-Kivi et al. [23] was the youngest (55 years) while that of the longest-lived patients was by Franceschi et al. [15] (63.25 years).

With respect to the involvement of the dominant arm, seven [15–17, 22–25] studies reflected whether it corresponds or not. In all of them, the involvement of the dominant arm was more common, it was 72.37% of the study sample, the highest frequency being 82% [22] and the lowest 56% [23].

### Diagnostic methods

The techniques used for medical diagnosis of dysfunctions were as follows: magnetic resonance imaging (MRI) in 11/11 of selected articles [15–25], arthroscopy in 10/11 [15–21, 23–25], radiography (RX) in 6/11 [15, 16, 19–22], physical examination (e.g., speed test) in 3/11 [19, 23, 24], ultrasound (US) in 1/11 [19]. Three articles [17, 18, 25] used MRI and arthroscopy, other [22] employed MRI and RX, four others [15, 16, 20, 21] used RX, MRI, and arthroscopy, two [23, 24] used physical examination, MRI and arthroscopy, and finally one paper [19] employed physical examination, RX, MRI, US, and arthroscopy.

### Dysfunctions and injury frequency

All papers included RC tears. Five [15, 18, 19, 23, 25] studies based their data on a classification according to the tear size (massive, large, medium, and small). In two [18, 25], the tear was medium or small, in another two [15, 19], there were as follows: large in 33% [15] and 19% [19]; medium in 36% [15] and 33% [19]; and small in 30% [15] and 47% [19], and in [23] the tear was small in 35%, medium in 25%, large in 24%, and massive in 14%.

On the other hand, two papers [15, 17] cataloged the tears according to their shape. The crescent-shaped tears were present in 49% [15] and 48% [17]; L shaped in 28% [15] and 15% [17]; inverse L shaped in 13% [17]; U shaped in 22% [15] and 2% [17]; and V shaped in 19% [17] tears. Likewise, four articles [15–17, 22] mentioned the tendons involved. In the case of a single tendon, the supraspinatus was affected in 100% [22], 58% [15], 54% [17], and 40% [16]. If the tear involved two tendons, the association of supraspinatus tendon (ST) and infraspinatus tendon (IT) was found in 41% [15], 13% [16], and 45% [17]; and that of ST and subscapularis tendon (SbT) in 18% [16]. Finally, the three tendons (ST, IT, and SbT) were affected in 27% [16].

In relation to LHBT, the dysfunctions observed were as follows: LHBT tear in 36% [16], 65% [18], 68% [19], 50% [20], 66% [21], 20% [22], 32% [23]; subluxation or dislocation in 31% [16], 34% [18], 20% [19], 34% [20], and 12% [23]; instability in 31% [16] and 65% [22]; type II SLAP in 100% [15], 59% [20], 12% [21], and 11% [23]; type II or type IV SLAP in 23% [19]; biceps pulley lesion in 34% [20]; tenosynovitis in 21% [21]; and severe inflammation of LHBT in 26% [19] and 43% [23]. Note the difference between type II SLAP, detachment of the superior labrum and biceps tendon from the glenoid rim, and type IV SLAP, and extension of the displaced bucket-handle labral tear into the biceps [26].

### Interventions

In relation to the *surgical treatment* applied to restore RC and LHB dysfunctions, all trials [15–25] used the arthroscopy technique, apart from two [23, 24] in which, in addition to arthroscopy, open surgery was performed.

Seven papers [15–18, 22, 24, 25] specified the technique used in the RC dysfunctions. All of them [15–18, 22, 24, 25] used suture anchors; three [16, 17, 24] out of seven used metallic anchors and two [15, 22] used biodegradable anchors. The techniques employed were single-row [15–18, 25], double-row [15–17, 25] and transosseous repair [18].

The LHB surgical treatment methods performed were SLAP repair [15], tenotomy [15–19, 21–23, 25] or tenodesis [15–25]. Ten [15–22, 24, 25] of the included trials employed arthroscopic tenodesis and two [23, 24] used open-surgery tenodesis.

Seven authors [16, 18, 19, 21–23, 25] compared tenotomy with tenodesis. Two trials [20, 24] contrasted two different tenodesis techniques. One [17] looked for differences between two techniques for RC dysfunctions. One [15] comparatively assessed SLAP repair and tenotomy.

Furthermore, *postoperative management* was reflected in 10/11 [15–20, 22–25] articles. The intervention consisted of a period of immobilization between 3 [17, 22], 4 [18, 20], and 6 weeks [15, 16, 20, 23–25]. During this time, passive mobilizations were carried out, specifically, in external rotation in [16, 22]; pendulum exercises were allowed, starting from the first post-operative day in [22] and in [20] shrugging both shoulders; active elbow, forearm, and hand and wrist motion were encouraged immediately after surgery. At 6 weeks, the sling was removed and overhead stretching with a rope and pulley was started in [15, 16]. In [18], pulley exercises were prescribed to increase their range of flexion. In [17, 23, 24], a ROM exercise program was started, followed by a muscle strengthening program using closed kinetic chain [17], exercises for RC, biceps, deltoid, pectoralis major, and scapular stabilizers. Muscle strengthening exercises were started at 9 to 12 weeks postoperatively in [20]. In [16], isotonic strengthening and rehabilitation of the RC, deltoid, and scapular stabilizers were initiated at 10 or 12 weeks after operation.

Rehabilitation was continued for 6 months and heavy manual work and overhead activities were allowed after 6 to 10 months after surgery in all papers [15–20, 22–25].

### Outcome measures and results

The measurements taken in the papers were in descending order of frequency: absence or presence of Popeye’s sign in 7/11 [18–23, 25]; pain in 6/11 [18–20, 22–24] using the visual analog scale (VAS) [18–20, 22, 24], and the numerical rating scale (NRS) [23]; muscle strength in 5/11 [17–19, 21, 22] measured with a digital dynamometer [17, 19, 21, 22] and with a digital force gauge transducer [18]; patient satisfaction in 4/11 [19, 23–25] based on VAS [23, 24] and measured with a question about the degree of satisfaction [19] and with a 5-point Smiley Scale [25]; ROM in 3/11 [15, 16, 20] measured with a standard universal goniometer; biceps brachii cramping in 2/11 [22, 23] assessed by means of an ultrasonographic evaluation [22] or the frequency of patient complaints [23]; and quality of life in 1/11 using SF-36 Health Survey [22]. All these parameters were measured independently. After surgery, general shoulder functionality was assessed using: Constant scale, 9/11 [17–25]; Simple Shoulder Test (SST), 4/11 [20, 21, 23, 24]; University of California at Los Angeles Shoulder Score (UCLA), 2/11 [15, 16]; American Shoulder and Elbow Surgeons (ASES), 2/11 [18, 20]; Disabilities of Arm, Shoulder and Hand (DASH), 1/11 [17, 25]; Korean Shoulder Scoring system (KSS), 1/11 [20].

Only five studies [15, 16, 18, 23, 25] compared the pre- and post-intervention of each experimental group (Table 4). The measured parameters were ROM, pain, and patient satisfaction. Related to the functionality, Constant, UCLA, SST, and ASES scales were used. Two articles [15, 16] found significant differences in each intervention. Each group showed a statistically significant improvement in ROM and UCLA.

**Table 4:** Outcome measures and results: significance and effectiveness of interventions

	Intervention groups	Pain	ROM	MS		Constant scale	UCLA	ASES	SST	DASH	KSS	PS	DS	Biceps cramps	SF-36
				Sh	Elb										
Franceschi et al. [15]	EG1: Art. for RC repair and type II SLAP repair		✓*				✓*								
	EG2: Art. for RC repair and biceps tenotomy		✓*				✓*								
Franceschi et al. [16]	EG1: Art. for RC repair and tenodesis without tenotomy of LHBT		✓*				✓*								
	EG2: Art. for RC repair and tenodesis with tenotomy of LHBT		✓*				✓*								
Lee et al. [18]	EG1: Art. for RC repair and LHBT tenotomy	✓*				✓*		✓*							
	EG2: Art. for RC repair and LHBT tenotomy and tenodesis	✓*				✓*		✓*							
Mardani-Kivi et al. [23]	EG1: Art. for RC repair and arthroscopic tenotomy of LHBT	✓*				✓*			✓*				✓*		
	EG2: Art. for RC repair and tenotomy + open subpectoral tenodesis of LHBT	✓*				✓*			✓*				✓*		
Van Deurzen DFP et al. [25]	EG1: Art. for RC repair and tenotomy of LHBT.					✓*									
	EG2: Art. for RC repair and LHBT tenotomy and tenodesis.					✓*									

✓= Parameter measured; *= significant and effective

Abbreviations: *Art*., arthroscopy; *ASES*, American Shoulder and Elbow Surgeon; *DASH*, Disabilities of Arm, Shoulder and Hand; *DS*, Degree of Satisfaction; *Elb*, elbow; *KSS*, Korean Shoulder Scoring system; *LHBT*, long head of biceps tendon; *MS*, muscle strength; *PS*, Popeye’s sign; *RC*, rotator cuff; *ROM*, range of motion; *SF-36*, SF-36 Health Survey; *Sh*, shoulder; *SST*, Simple Shoulder Test; *UCLA*, University of California at Los Angeles Shoulder Score

One [18] obtained significant improvements in pain, in Constant, and in ASES scales in every experimental group. Another one [23] found significant differences in each intervention in Constant, SST, pain, and satisfaction. Both groups improved these mentioned parameters. One [25] showed substantial improvements in Constant Score in both groups. Table 4 summarizes the significance and effectiveness of interventions of these 5 studies.

On the other hand, in relation to the comparison between the interventions of all selected studies (Table 5), five [15, 18, 21–23] achieved changes in some of their parameters. For the most frequently considered parameters, the studies with significant outcomes and effective were as follows: 100% for biceps cramping [22, 23], 57% for Popeye’s sign [18, 21–23], 50% for UCLA [15], 33% for ROM [15], 25% for patient satisfaction [23], and 20% for forearm supination power [18].

**Table 5:** Outcome measures and results: comparison of the effectiveness of the studies interventions

	Pain	ROM	MS		Constant scale	UCLA	ASES	SST	DASH	KSS	PS	DS	Biceps cramp	SF-36
			Sh	Elb										
Franceschi et al. [15]		✓*				✓*								
Franceschi et al. [16]		✓				✓								
Grasso et al. [17]			✓		✓				✓					
Lee et al. [18]	✓			✓*	✓		✓				✓*			
Zhang et al. [19]	✓			✓	✓						✓	✓		
Park et al. [20]	✓	✓			✓		✓	✓		✓	✓			
De Carli et al. [21]			✓	✓	✓			✓			✓*			
Castricini et al. [22]	✓		✓	✓	✓						✓*		✓*	✓
Mardani-Kivi et al. [23]	✓				✓			✓			✓*	✓*	✓*	
Mardani-Kivi et al. [24]	✓				✓			✓				✓		
Van Deurzen et al. [25]					✓						✓	✓		

✓= parameter measured; *= significant and effective

Abbreviations: *ASES*, American Shoulder and Elbow Surgeon; *DASH*, Disabilities of Arm, Shoulder and Hand; *DS*, degree of satisfaction; *Elb*, elbow; *KSS*, Korean Shoulder Scoring system; *MS*, muscle strength; *PS*, Popeye’s sing; *ROM*, range of motion; *SF-36*, SF-36 Health Survey; *Sh*, shoulder; *SST*, Simple Shoulder Test; *UCLA*, University of California at Los Angeles Shoulder Score

Specifically, in [15], group II intervened through biceps tenotomy and RC repair and showed statistically significantly better results in UCLA scores and ROM than group I, which operated through type II SLAP repair and RC repair. In [18], group II intervened through tenodesis with tenotomy showed greater forearm supination power than group I, which operated through tenotomy (*p* = 0.02). In [21], the incidence of Popeye’s sign was significantly higher in group II, operated through tenotomy, than group I, intervened through tenotomy and tenodesis. In [22], the incidence of Popeye’s sign was significantly higher in group I of tenotomy than in group II of tenodesis, while the incidence of biceps brachii cramping was higher in the tenodesis (*p* = 0.043). In another one [23], Popeye’s sign and biceps brachii cramping were significantly higher in group I of arthroscopic tenotomy than in group II of open subpectoral tenodesis, and patient satisfaction was significantly higher in tenodesis.

The clinical interventions of the included studies [15–25] improved the parameters addressed in this section, although no significant differences were found between both groups. Table 5 summarizes these data.

## Discussion

This systematic review compiled the randomized clinical trials that included subjects with lesions diagnosed for RC and LHB, identifying and analyzing surgical and post-surgical approaches, if the latter was specified, as well as their efficacy on associated dysfunctions on both structures. Thus, sample data, diagnostic methods, dysfunctions and injury frequency, interventions, outcome measures, and results obtained were extracted. Secondarily, the functional evaluation methods on associated dysfunctions on RC and LHB were analyzed.

Regarding the methodological quality, this systematic review required randomized clinical trials to score at least 6 out of 10 on the PEDro scale [13], i.e., *good* methodological quality. It is noteworthy that 3 out of 10 scoring items are related to blinding (5, 6, and 7), which is difficult to meet in the surgical and post-surgical procedures addressed [27]. Thus, these items did not score particularly well. In particular, the blinding of clinicians who administered the therapy did not score any points. However, regarding the patients, three of the included studies [18, 20, 22] were blinded despite the handicap. The methodological quality of Castricini et al. [22] was even assessed as *excellent* since, in addition to the patients, it blinded the evaluators of the intervention. Thus, said author ensured that the results of the interventions were not conditioned by the subjects and also decreased the probability of clinical trial bias.

Since the PEDro scale determines the probability of the result validity and that it contains sufficient information to guide clinical practice [13], the good methodological quality of the selected studies evidenced these attributes. This is complemented by the evidence levels (I and II), determined by the authors of the included studies themselves.

In relation to the sample, RC and LHB dysfunctions can occur at any age, although the mean of these studies is around 60 years, consistent with the reduction of the blood supply characteristic of age [28], which contributes to tendon degeneration [29]. As for sex, there are no major differences between men and women. This equity is refuted in the study of Razmjou et al. [30] in which the associated lesions affected the male population more significantly. However, Lee et al. [18], in which the study sample was consecutive, the high incidence percentage of women (77%) may be due to the increase of calcium and/or hydroxyapatite deposits on tendons because of the hyperparathyroidism in menopausal women [31]. Likewise, seven studies [15–17, 22–25] mentioned the involvement of the dominant arm, this being the injury with a large percentage, surpassing 80% [22, 24] of cases. This high incidence is due to the degeneration of RC tendons because of the overuse of the dominant arm in activities of daily life. This explains what happens more in workers and athletes [11] whose requirements are greater.

With respect to the methods of evaluation and diagnosis, some clinicians use special tests for RC tears [32] and for biceps [23]. In addition to these tests, it is convenient to use complementary imaging tests rather than giving additional information, thus obtaining, a more accurate diagnosis. In relation to this review, all the analyzed studies have used complementary imaging tests. The most commonly used was MRI, since it allows to recognize factors of poor prognosis, such as tedious retraction, atrophy, and fat infiltration of the muscular tummy, and identify lesions associated to the glenohumeral joint [33]. Likewise, Iannotti et al. [33] described a sensitivity of MRI of 89% and a specificity of 100% in the detection of rotator tears not subjected to previous surgery. Ardic et al. [34] show MRI and US had a comparable high accuracy for identifying the biceps pathologies and RC tears. Despite this, MRI was superior to ultrasonography in many shoulder structures [34]. Nevertheless, MRI is still a costly method [35] of somewhat limited availability and may be contraindicated for medical reasons or for claustrophobia [36].

On the other hand, the relationship between RC and LHB was proposed. The associated pathologies were RC tears and LHBT tears, subluxations, or dislocations. In the case of a single injured tendon, the supraspinatus is the most frequently affected [18] and it is associated with partial or total tears of biceps tendon. However, when the injury affects the SbT, it is very probable that there is an instability-like dysfunction of the LHB, due to the intimate morpho-functional relationship existing between the structures in relation to the reflex pulley [37] charged with maintaining the integrity of LHB. In the case of an involvement of 2 tendons, the association of ST and IT is the most commonly related to LHB tendinopathy [7].

As for the surgical treatment applied to restore RC dysfunctions, all trials [15–25] used the arthroscopy technique. Compared with traditional open techniques, arthroscopic repairs offer patients smaller incisions and less soft-tissue trauma, which result in improved postoperative pain and rehabilitation [38]. Besides, seven papers [15–18, 22, 24, 25] used suture anchors in RC lesion. Regarding the type of suture anchor, three [16, 17, 24] used metallic anchors and two [15, 22] biodegradable anchors. As for the anchor composition, each material has its inherent advantages and disadvantages. For example, metallic suture anchors are opaque in X-rays and are hence easily evaluated in the clinical setting. However, these pose challenges in revision RC surgery [38], including distortion of preoperative MRI and necessity of removal before a new anchor can be put in place [38]. On the other hand, bio-absorbable anchors are radiolucent in X-rays and have less distortion on MRI [38]. Nevertheless, there are cases of an exaggerated immune response resulting in significant osteolysis, chondrolysis, and premature anchor failure [38].

Regarding LHB surgical treatment, there is no consensus on which technique is the most effective, since some authors, such as Walch et al. [39], defended tenotomy as producing more satisfactory results, while others, such as Checchia et al. [40], also obtained results in patients treated with LHB tenodesis. However, tenodesis seems to prevent complications associated with tenotomy, such as Popeye’s sign, atrophy, and muscle weakness [41]. This fact is corroborated in this review, in which the incidence of Popeye’s sign is significantly higher in the tenotomy group [18, 21–23].

As obtained in the results of this study, ten [15–20, 22–25] trials used a postoperative rehabilitation in addition to the surgical one.

In relation to this postoperative intervention, physiotherapy currently addresses shoulder dysfunction through numerous procedures [42–44], in accordance with authors such as Kuhn et al. [45]. This study [45] considers joint mobilizations, massage, transcutaneous electrical stimulation nerve stimulation, US, laser treatment, extracorporeal shockwave [46], and a flexibility and strength exercise program [45, 47]. Scientific evidence demonstrates that the combination of exercise and manual therapy significantly decreases pain and increases functionality [45]. Therefore, the selected trials [15–20, 22–25], included flexibility and strength exercise programs and joint mobilizations performed from the first day in order to increase recovery speed and decrease the period of disability [48]. The role of the scapula in omalgia is considered especially relevant nowadays due to moving the upper extremity through its greatest ROM, the shoulder complex (thorax, humerus, and scapula) must work in a synchronized manner [49]. Thus, optimal shoulder functionality is directly related to adequate scapular biomechanics [50]. For this reason, some authors such as Struyf et al. [46], Moezy et al. [51], and Baskurt et al. [52] add, to the rehabilitation of shoulder pathology, scapular stabilization exercise programs [15–18, 53].

To assess treatment efficacy, the authors measured pain, ROM, muscle strength, absence or presence of Popeye’s sign, biceps brachii cramping, quality of life, and degree of satisfaction separately. The sum of the mentioned parameters, except for Popeye’s sign, biceps cramping, and quality of life, involves the way the global functionality of the shoulder is addressed. Even so, every study used functional rating scales that offered a single score, even if independently rated items had been included. All clinical interventions from the included studies [15–25] improved those parameters separately, as well as global functionality through rating scales, so any intervention of associated dysfunctions on the RC and LHB is effective from a clinical perspective. As to the shoulder rating scales used, the authors mainly used the Constant Score as a functional assessment method to determine the significant differences. This scale is employed in shoulder pathologies, especially RC [54, 55]. Its frequent use is because it is easy to complete and interpret [55] and it is also extremely useful in monitoring RC dysfunctions [55].

Regarding the strengths of the study, it should be noted that randomized clinical trials were only included to minimize the risk of review bias, in addition to a requirement of a minimum of 6 points on the PEDro scale, that is, that the methodological quality was *good*. On the other hand, the search was carried out with no time limit (i.e., without applying any time filter in the databases), to obtain more scientific evidence, as well as assess the new healthcare trends. Finally, this review provides detailed information on the functional assessment tools used by every author. This highlights the importance of these methods, which contain both objective and subjective data from clinicians and patients themselves (patient-reported outcome measures).

Regarding the limitation of the study, the authors considered that the surgical techniques used in the studies, as well as the items addressed and the measurement systems applied, were broadly heterogeneous, thus preventing any meta-analysis. On the other hand, one of the included articles [21] did not specify the physiotherapeutic intervention in detail. Based on this, the authors propose a review that focused its attention on the physiotherapeutic protocol used in the combined impacts of RC tendons and LHBT prospectively. After analyzing the results obtained in this study, a new systematic review addressing “the efficacy of treatment on RC and superior labral dysfunctions” could be of interest. We also consider more randomized clinical trials where surgical techniques and associated conservative clinical procedures are homogenized to be necessary (e.g., corticosteroid injections or physiotherapy) in order to obtain robust evidence of the effectiveness of the interventions. This would enable the creation of quality clinical action protocols.

In conclusion, this systematic review analyzed the efficacy of surgical and post-surgical interventions used, when the latter were specified, in the joint dysfunctions of the tendons attached to the RC and the LHBT.

All the approaches in general, surgical plus post-surgical, were always effective in relation to the parameters measured in each study. These, arranged by order of frequency, were functionality, Popeye’s sign, pain, strength, ROM, satisfaction, biceps brachii cramping, and quality of life.

Regarding the surgical interventions used, all papers considered an arthroscopic approach, as it is a minimally invasive technique and therefore advantageous in subsequent recovery. Only a few of them also included open surgery. The techniques used in the restoration of the RC lesion, arranged by decreasing order of frequency, were as follows single-row, double-row, and transosseous repair. Regarding the LHB, the vast majority of authors employed tenodesis followed immediately by tenotomy, and only one used SLAP II repair. Trials comparing tenotomy with tenodesis showed that tenodesis had better results than tenotomy, with a statistically significant difference in Popeye’s sign, satisfaction, and forearm supination strength. However, there was no difference regarding biceps cramping.

As regards post-surgical treatment, except for one author who did not consider this intervention, the studies included a period of immobilization, passive and active mobilizations, stretching, and muscle strength exercise programs. In this respect, considering physiotherapy treatment after surgery in the studies reflects a broad clinical interest by researchers in tackling shoulder injuries in a multidisciplinary manner, always for the sake of the patient’s functional recovery.

All studies measure the shoulder functionality using functional assessment scales. The tools used, and ranked from highest to lowest use, were Constant Scale, SST, UCLA, ASES, DASH, and KSS. These scales are a fundamental clinical element, because they afford specialists a greater degree of objectivity and also unify the language among professionals, in order to achieve an effective approach of RC and LHBT injuries.

## Supplementary Information

**Additional file 1.**

## Data Availability

Not applicable.

## Abbreviations

ASES: American Shoulder and Elbow Surgeons
DASH: Disabilities of Arm, Shoulder and Hand
IT: Infraspinatus tendon
KSS: Korean Shoulder Scoring system
LHB: Long head of the biceps
LHBT: Long head of the biceps tendon
MRI: Magnetic resonance imaging
NRS: Numerical rating scale
RC: Rotator cuff
ROM: Range of motion
RX: Radiography
SbT: Subscapularis tendon
SD: Standard deviation
SST: Simple Shoulder Test
ST: Supraspinatus tendon
UCLA: University of California at Los Angeles Shoulder Score
US: Ultrasound
VAS: Visual analog scale
